# Gastrointestinal Hemorrhage From Esophageal Dissecans Superficialis After the Initiation of a Tyrosine Kinase Inhibitor

**DOI:** 10.7759/cureus.16471

**Published:** 2021-07-19

**Authors:** Gowthami Kanagalingam, Vanessa Sostre Santiago, Vrinda Vyas, Divey Manocha

**Affiliations:** 1 Internal Medicine, State University of New York Upstate Medical University, Syracuse, USA; 2 Gastroenterology, State University of New York Upstate Medical University, Syracuse, USA

**Keywords:** gastrointestinal hemorrhage, upper endoscopy, immunotherapy, esophagus, cancer

## Abstract

Esophagitis dissecans superficialis (EDS) is described as the peeling of squamous mucosa of the esophagus with regurgitation of esophageal casts. It is a rare endoscopic finding associated with chemical irritants, autoimmune disorders, or medications. Most patients are asymptomatic but clinical manifestations can include dysphagia, heartburn, bleeding, or vomiting. In this report, we present a case of a 70-year-old man with a previous history of small cell lung and hepatocellular carcinoma for which he had undergone chemoradiation and immunotherapy. He had presented with upper gastrointestinal hemorrhage manifested as coffee ground emesis. Endoscopic findings were consistent with EDS. No recurrence of his gastrointestinal hemorrhage was observed after acid-suppressive therapy. With our case report, we aim to increase awareness for EDS as a differential diagnosis for gastrointestinal hemorrhage.

## Introduction

Esophagitis dissecans superficialis (EDS) or sloughing esophagitis is a rare and benign disorder which is characterized by the sloughing of the esophageal mucosa [1]. EDS was first described by Dr. L. Rosenberg in 1892 as peeling of the squamous mucosa of the esophagus with regurgitation of esophageal casts [2]. An incidence of 0.03% has been reported; it predominantly affects adults > 50 years old [3,4]. Most cases of EDS are idiopathic. Other causes include heavy smoking, hot beverages, achalasia, celiac disease, medications (nonsteroidal anti-inflammatory drugs, potassium chloride, bisphosphonates), and esophageal iatrogenic (band ligation, mediastinal radiation, bullous dermatosis, immunosuppression, impaired mobility) {5}. We present an older man who presented with upper gastrointestinal hemorrhage. He was found to have esophagitis dissecans on endoscopy after recently being started on a tyrosine kinase inhibitor. 

## Case presentation

A 70-year-old man with a past medical history of stage IIIa squamous cell carcinoma of the lung with concurrent metastatic hepatocellular carcinoma Barcelona Clinic Liver Cancer (BCLC) stage D, compensated cirrhosis secondary to untreated chronic hepatitis C, and cerebrovascular accident with mild left-sided residual deficits presented to the hospital with multiple episodes of coffee ground emesis for one day. He denied dysphagia, odynophagia, chest pain, melena or hematochezia.

The patient had been diagnosed with squamous cell carcinoma of the lung and hepatocellular carcinoma for which he underwent concurrent chemotherapy (with carboplatin and taxol), ablation, and radiation therapy. He was then initiated on lenvatinib, a tyrosine kinase inhibitor, four months before presentation. Lenvatinib was discontinued a few days prior to admission due to fatigue, loss of appetite, weight loss, nausea, and intermittent diarrhea.

He did not have any known allergies. The patient was a former smoker without alcohol use. He had a normal colonoscopy five years ago but no previous upper endoscopy was done. His medications on admission included aspirin, lisinopril, morphine, and melatonin

On admission, the patient was hemodynamically stable and afebrile. Body mass index was 17.8 kg/mᒾ. Clinically, the patient looked frail and cachectic. The abdomen was mildly distended with no tenderness, rebound, or guarding. Bowel sounds were normal. Blood work on admission is shown in Table 1. 

**Table 1:** Blood work on admission BUN: Blood urea nitrogen; ALP: Alkaline phosphatase; AST: Aspartate aminotransferase; ALT: Alanine aminotransferase; INR: International normalized ratio

	Value	Reference
Hemoglobin	8.6 g/dl	13.5 - 18g/dl
White blood cell count	11.7 x10*3	4 - 10 x10*3
Platelet count	115 x10*3	150 - 400x 10*3
Creatinine	0.84 mg/dl	0.7 - 1.20mg/dl
Sodium	134 mmol/l	136 - 145mmol/l
BUN	60 mg/dl	6 - 20 mg/dl
Albumin	3 g/dl	3.5 - 5.2g/dl
Total bilirubin	9.4 mg/dl	< 1.2 mg/dl
Direct Bilirubin	7.1 mg/dl	<0.3 mg/dl
ALP	759 U/l	40 - 129 U/l
AST	603 U/l	<40 U/l
ALT	305 U/l	<41 U/l
Lipase	16 U/l	7 - 60 U/l
INR	1.12	0.8 - 1.1

**Figure 1:**
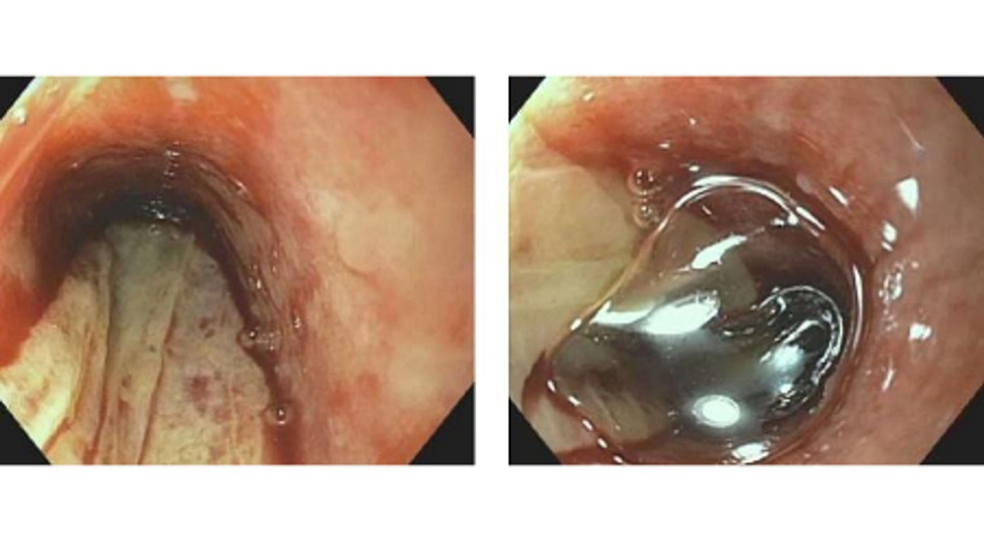
White vertical strip of squamous mucosa detached from the esophagus consistent with esophageal dissecans superficialis, moderately severe erosive esophagitis, and radiation esophagitis

Pantoprazole, octreotide drip, and prophylactic antibiotic therapy with ceftriaxone were initiated. He then underwent an esophagogastroduodenoscopy which revealed a white vertical strip of squamous mucosa detached from the esophagus consistent with EDS, moderately severe erosive esophagitis, and radiation esophagitis (Figure 1). Histopathology showed fibrinopurulent exudate and rare reactive squamous cells consistent with ulceration. There was no evidence of bacterial or fungal infection. The patient was then switched to pantoprazole 40 mg BID and sucralfate. No recurrence of gastrointestinal bleeding was noted.

## Discussion

The pathogenesis of EDS is unknown but possible allergic responses or insults such as chemical, physical, immunological or thermal injury are proposed [5]. Purdy et al. noted that patients with EDS were older and more debilitated, such as patients from nursing homes, requiring oxygen, status post organ transplant, immunosuppression, metastatic cancer, and malnutrition. Further, patients with EDS were more likely to be on five or more medications [6]. 

The cause for EDS in our patient was likely multifactorial. He had multiple underlying comorbidities such as small cell lung cancer, hepatocellular carcinoma, and compensated cirrhosis which placed him at an increased risk. Further, treatment for his concurrent small cell lung cancer and HCC with chemoradiation therapy was likely contributory. With his underlying history of cerebrovascular accident with residual weakness, he was also prone to decreased mobility. An additional risk factor was his malnutrition which likely resulted in the setting of his malignancy and from side effects from targeted therapy with lenvatinib. Lenvatinib is a tyrosine kinase inhibitor that inhibits fibroblast growth factor (FGF) receptors -14, vascular endothelial growth factor (VEGF) receptors 1-3, KIT, RET, and platelet-derived growth factor (PDGF) receptor alpha. Lenvatinib is indicated for patients with good performance status, Child-Pugh A class, and BCLC-C without invasion of the main portal vein, patients with tumor progression are not suitable for loco-regional therapies [7].

Patients with EDS are usually asymptomatic but can present with dyspepsia, dysphagia, odynophagia, heartburn, regurgitation, weight loss, and upper gastrointestinal bleeding [8]. In the REFLECT trial, the most common adverse events of lenvatinib therapy included hypertension, decreased appetite, weight loss, and diarrhea [9]. Our patient presented with upper gastrointestinal bleeding but had noted decreased appetite, weight loss, nausea, and vomiting for the preceding two months since starting lenvatinib. This could be explained as side effects from targeted therapy with lenvatinib or in the setting of his EDS, erosive esophagitis, and radiation esophagitis. Thus far no reports of esophagitis under lenvatinib therapy have been reported. It is therefore unclear if lenvatinib played a role in the development of this patient's EDS. We suspect that this targeted therapy with his underlying conditions placed him at an increased risk for EDS. 

Endoscopic features include single or multiple white patches of sloughing mucosa which are found in the lower ⅔ of the esophagus or diffuse distribution. The endoscopic findings are called gift wrap ribbons. Histopathological findings include tubular white casts from sloughed squamous mucosa and intraepithelial cleft with strips of squamous epithelium in two tones. The superficial sloughed layer is characterized by parakeratosis or coagulative necrosis whereas the squamous mucosa below the split shows basal zone expansion or can have a normal appearance. None or very limited inflammation is usually seen. Endoscopic findings can be similar to eosinophilic esophagitis or candida esophagitis. Differential diagnosis includes pill esophagitis, candidiasis, celiac disease, mucosal trauma, artifact, and rigid endoscopy [3,10-12]. 

Hart et al. proposed the following criteria for the diagnosis of EDS: strips of sloughed esophageal mucosa > 2 cm long, normal underlying esophageal mucosa, without friability or ulcerations on adjacent mucosa [13]. EDS is a self-limited disorder. There are no specific recommendations for treatment or follow up. Elimination of the risk factors and acid suppression has been proposed [1,7,11].

## Conclusions

EDS should be an important differential diagnosis in patients presenting with gastrointestinal hemorrhage, especially with multiple comorbidities, immobilization, and immunosuppression. To the best of our knowledge, there are only a few case reports of patients with EDS presenting as gastrointestinal hemorrhage and this is the first case of a patient with EDS after initiation of a tyrosine kinase inhibitor. With our case report, we aim to increase awareness for this underreported and under-diagnosed entity as a differential diagnosis for gastrointestinal hemorrhage.

## References

[1] Orosz E, Patel AV (2021). Sloughing mucosa in esophagitis dissecans superficialis. Clin Gastroenterol Hepatol.

[2] Nasir UM, Rodgers B, Panchal D, Choi C, Ahmed S, Ahlawat S (2020). Ferrous sulfate-induced esophageal injury leading to esophagitis dissecans superficialis. Case Rep Gastroenterol.

[3] Carmack SW, Vemulapalli R, Spechler SJ, Genta RM (2009). Esophagitis dissecans superficialis ("sloughing esophagitis"): a clinicopathologic study of 12 cases. Am J Surg Pathol.

[4] Fiani E, Guisset F, Fontanges Q, Devière J, Lemmers A (2017). Esophagitis dissecans superficialis: a case series of 7 patients and review of the literature. Acta Gastro-Enterologica Belgica.

[5] Akhondi H (2014). Sloughing esophagitis: a not so common entity. Int J Biomed Sci.

[6] Purdy JK, Appelman HD, McKenna BJ (2012). Sloughing esophagitis is associated with chronic debilitation and medications that injure the esophageal mucosa. Mod Pathol.

[7] European Association for the Study of the Liver (2018). EASL Clinical Practice Guidelines: management of hepatocellular carcinoma. J Hepatol.

[8] Rokkam VR, Aggarwal A, Taleban S (2020). Esophagitis dissecans superficialis: malign appearance of a benign pathology. Cureus.

[9] Kudo M, Finn RS, Qin S (2018). Lenvatinib versus sorafenib in first-line treatment of patients with unresectable hepatocellular carcinoma: a randomised phase 3 non-inferiority trial. Lancet.

[10] Lamine H, Bochra B, Mouna M, Heykel E, Monia T, Mohamed Masaddak A (2018). Esophagitis dissecans superficialis due to severe nonsteroidal anti-inflammatory drugs toxicity. Presse Med.

[11] Brownschidle SS, Ganguly EK, Wilcox RL (2014). Identification of esophagitis dissecans superficialis by endoscopy. Clin Gastroenterol Hepatol.

[12] Panarelli NC (2017). Other forms of esophagitis: it is not gastroesophageal reflux disease, so now what do I do?. Surg Pathol Clin.

[13] Hart PA, Romano RC, Moreira RK, Ravi K, Sweetser S (2015). Esophagitis dissecans superficialis: clinical, endoscopic, and histologic features. Dig Dis Sci.

